# Iron overload by Superparamagnetic Iron Oxide Nanoparticles is a High Risk Factor in Cirrhosis by a Systems Toxicology Assessment

**DOI:** 10.1038/srep29110

**Published:** 2016-06-30

**Authors:** Yushuang Wei, Mengzhu Zhao, Fang Yang, Yang Mao, Hang Xie, Qibing Zhou

**Affiliations:** 1 Department of Nanomedicine & Biopharmaceuticals, National Engineering Research Center for Nanomedicine, Huazhong University of Science and Technology, Wuhan, Hubei, China; 2 Division of Viral Products, Office of Vaccines Research and Review, Center for Biologics Evaluation and Research, United States Food and Drug Administration, Silver Spring, Maryland, United States of America

## Abstract

Superparamagnetic iron oxide nanoparticles (SPIONs) as a contrast agent have been widely used in magnetic resonance imaging for tumor diagnosis and theranostics. However, there has been safety concern of SPIONs with cirrhosis related to excess iron-induced oxidative stress. In this study, the impact of iron overload by SPIONs was assessed on a mouse cirrhosis model. A single dose of SPION injection at 0.5 or 5 mg Fe/kg in the cirrhosis group induced a septic shock response at 24 h with elevated serum levels of liver and kidney function markers and extended impacts over 14 days including high levels of serum cholesterols and persistent low serum iron level. In contrast, full restoration of liver functions was found in the normal group with the same dosages over time. Analysis with PCR array of the toxicity pathways revealed the high dose of SPIONs induced significant expression changes of a distinct subset of genes in the cirrhosis liver. All these results suggested that excess iron of the high dose of SPIONs might be a risk factor for cirrhosis because of the marked impacts of elevated lipid metabolism, disruption of iron homeostasis and possibly, aggravated loss of liver functions.

Superparamagnetic iron oxide nanoparticles (SPIONs) are contrast enhancing agents used in the magnetic resonance imaging (MRI) for diagnosis of tumors and potentially theranostics for cancer treatment^1^,^2^. Lately, SPIONs have been used in the diagnosis of focal liver lesions and progression of fibrosis in the steatohepatitis by the assessment of levels of nanoparticles in Kupffer cells^3^,^4^. While the *in vitro* cell viability study indicates that the cytotoxicity of SPIONs is cell-specific and varies with the size of nanoparticles and types of coating materials^5^, high levels of excess iron have been persistently observed in liver tissues over weeks^6^,^7^,^8^,^9^. Despite that iron overload has been shown not to impair the liver function nor cause significant immunotoxicity responses in the health model^7^, the persistent excess amount of iron in the liver has been a safety concern due to the induced oxidative stress and elevated lipid peroxidation^7^,^8^,^9^,^10^,^11^. These safety concerns are often deepened by different clinical manifestations of cancers, for instance, cirrhosis in liver cancer that is characterized with fibrosis and decreased liver functions^12^,^13^. In fibrosis, lipid peroxidation induced by excess iron has been a serious toxicity issue in the non-alcohol related fatty liver because the liver is the major organ for lipid biosynthesis and metabolism^14^,^15^,^16^,^17^. Therefore, SPION-induced iron overload could potentially increase the risk of progression of cirrhosis patients considering the necroinflammatory environment (necrosis and inflammation) in the liver^15^,^17^. However, a systemic toxicity evaluation of SPIONs in cirrhosis has not been reported, and thus is highly needed for the assessment of the potential risk and mechanisms involved.

Systems toxicology is a new forging cross-disciplinary toxicology field that provides in-depth risk assessment of chemical entities in a biological system^18^. The systems toxicology approach has demonstrated hepatotoxicants and tobacco smoke toxicants-induced gene expression for prediction of the toxicity risk^19^,^20^. Microarray analysis has also been used in the systems toxicology to elucidate cytotoxic mechanisms of PPAR gamma agonist drugs^21^,^22^. In this study, a mouse cirrhosis model with the use of biocompatible SPIONs was assessed by the systems toxicity approach. The biocompatible SPIONs have recently been reported to produce high MRI contrast of liver tumor at a low dose and had low retention in the liver and spleen with full excretion through urine^23^. In this study, the impact of biocompatible SPIONs in cirrhosis was first determined by serum biochemistry profiles post SPION injection over fourteen days. The effect of SPIONS on the regulation of 370 genes from more than thirteen molecular toxicity pathways in the liver tissue was then assessed by RT^2^ PCR array. The overall toxicity assessment was achieved by the combination of these two approaches to reveal the underlying toxicity risk and mechanisms.

## Results

### Acute iron overload in the cirrhosis model post SPION injection

Biocompatible SPION solution was prepared as reported and used for the toxicity study due to its rapid *in vivo* clearance^23^. The hydrodynamic average size of SPIONs was 120.3 ± 2.4 nm with a zeta potential of −4.31 ± 0.13 mV (Supplementary Fig. S1). The TEM analysis confirmed the synthesized SPIONs as nanoclusters of ultrafine iron oxide nanoparticles as reported (Supplementary Fig. S1)^23^. Moreover, the synthesized SPIONs exhibited a high stability in the 10% serum buffered solution at 37 °C over 7 days with no significant changes of the zeta average size whereas the polydispersoin index increased initially to 0.18 at 24 h and then remained unchanged (Supplementary Fig. S1). Cirrhosis in the liver was induced based on the method reported with a successful rate of more than 90% 24. The presence of fibrosis was confirmed by Sirius red staining in the liver tissue in week six (Supplementary Fig. S2). Intravenous injection of the SPION nanocluster solution was first carried out at a single dose of 5 mg Fe/kg body weight. This milligram iron dose has been commonly used in the animal studies^6^,^7^,^8^. In addition, Feridex IV, the FDA approved clinical MRI contrast agent that has been discontinued by the manufacturer, has a prescribed dosage of 0.56 mg Fe/kg body weight for human that is equivalent to a dose of 6.9 mg Fe/kg for mouse based on the body surface area^25^. Furthermore, the toxicity study of the contrast agent in animals normally requires approximately 100 times the amount used for human according to US FDA’s guidelines^26^. At the dosage of 5 mg Fe/kg, we observed no obvious abnormal body weight loss or mortality in the cirrhosis or the normal mice over two weeks post SPION injection.

**Figure 1:**
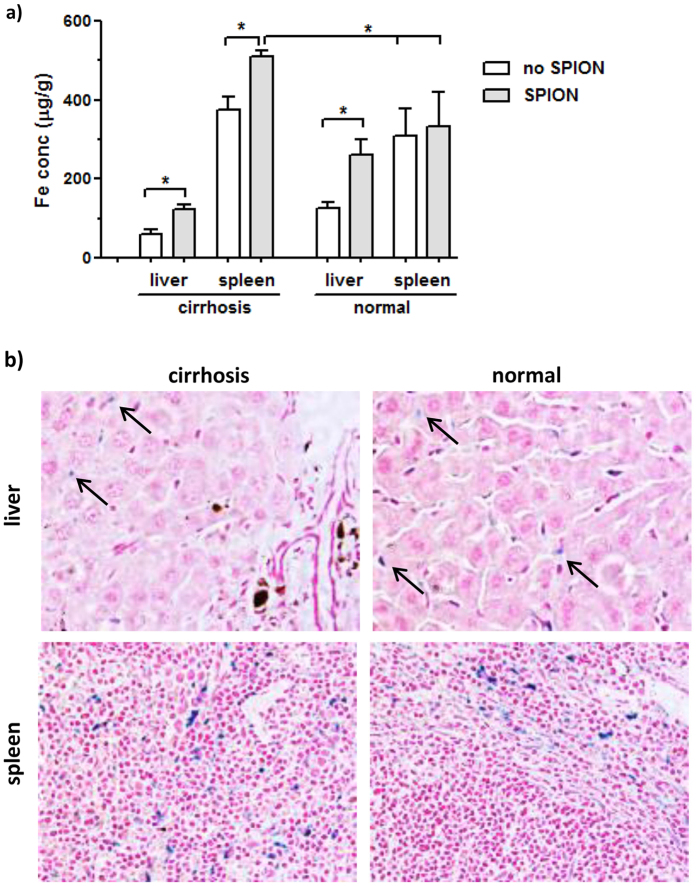
Distribution of excess iron in the liver and spleen tissues post the SPION injection in the cirrhosis and normal groups. (**a**) Levels of iron concentration were obtained at 24 h post injection and calculated as μg Fe per weight of the tissue samples. The data presented were the average ± SD of four samples in each group. Statistical analysis was performed with one way ANOVA and Tukey’multiple comparison test using the GraphPad Prism program (*P < 0.05). (**b**) Prussian blue staining of the presence of iron deposits (blue color indicated by arrows) in the pathological tissue slides at 24 h post SPION injection.

The levels of iron overload in the liver and spleen were determined at 24 h post SPION injection because these two organs were the major accumulation sites of SPIONs^6^,^7^,^8^,^9^. In the cirrhosis mice, the iron level increased to 122.3 ± 12.7 μg/g tissue weight post SPION injection as compared to 60.9 ± 5.2 μg/g without injection (Fig. 1a). In the spleen tissues, the iron level arose from 377.4 ± 33.7 μg/g to 511.3 ± 13.4 μg/g post SPION injection. In the normal mice, the basal iron levels were much higher at 126.4 ± 14.4 μg/g in the liver and reached 262.8 ± 38.6 μg/g post SPION injection. However, there was only a minor increase by 25 μg/g in the spleen post SPION injection (Fig. 1a). The increased iron levels in the spleen post SPION injection were consistent with histopathological Prussian blue staining of the iron deposits in the tissues (Fig. 1b). More iron positive deposits were found in the spleen tissue of the cirrhosis group, although the difference of positively stained Kupffer cells between the cirrhosis and the normal liver tissues (as indicated by arrows, Fig. 1b) was not obvious. In the cirrhotic liver, no other pathological damage was observed in the tissues at 24 h post SPION injection except the presence of the scar tissue at the right side (Fig. 1b), clearly the result of the damage induced by DDC.

Cirrhosis is often accompanied by an enlarged liver, and thus the weights of the whole organs were used to estimate the total amount of excess iron in the liver and spleen (Table 1). The average weight of the cirrhotic liver was found to be almost twice that of the normal one (P < 0.01, Table 1). Thus, although the iron levels in the cirrhotic liver were half of those of the normal liver (Fig. 1a), the total amount of excess iron in the cirrhotic liver accounted approximately 80% of the iron from SPION injected as compared to 91.8% in the normal liver (Table 1). Clearly, most of SPIONs injected was retained in the liver even with cirrhosis (Fig. 1b). On the other hand, there was no difference in the average weight of the spleens between the groups. The amount of excess iron in the spleen of the cirrhosis group was 11.9% of total iron injected versus only 2.2% in the normal group. This result indicated that cirrhosis did induce loss of liver functions and resulted in the overflow of 10% of injected iron into the spleen. Therefore, the serum biochemistry profiles post SPIO injection were investigated over a period of 14 days to determine the impact of iron overload.

### SPIONs induced both short-term and long-term changes of serum biochemistry markers

**Figure 2:**
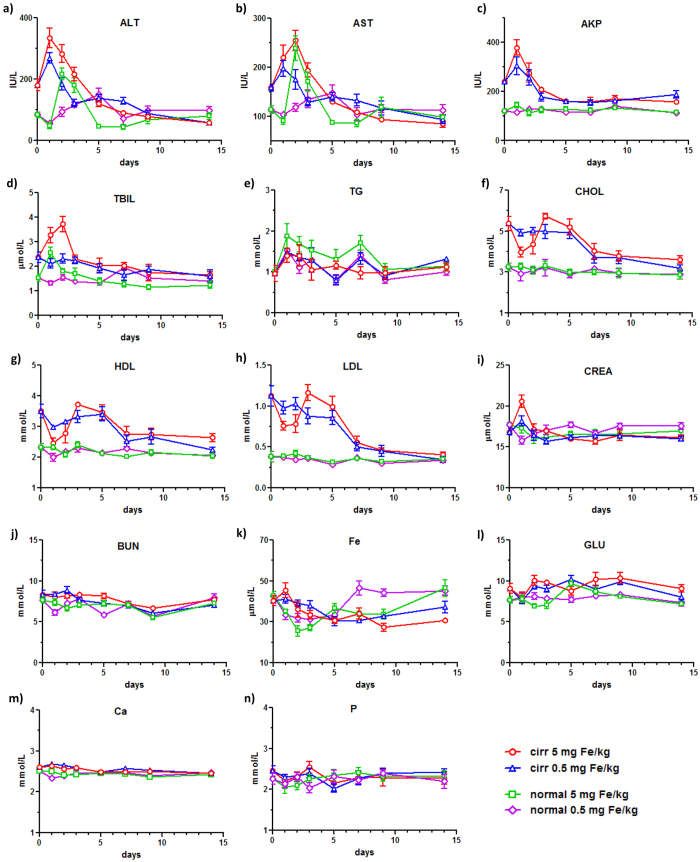
Levels of serum biochemistry profiles of cirrhosis and normal mice post SPION injection over 14 days. Each data point represented the mean ± SEM of serum samples from six mice. (**a**) ALT: alanine aminotransferase; (**b**) AST: aspartate aminotransferase; (**c**) AKP: alkaline phosphatase; (**d**) TBL: total bilirubin; (**e**) TG: triglycerides; (**f**) CHOL: cholesterol; (**g**) HDL: high-density lipoprotein cholesterol; (**h**) LDL: low density lipoprotein cholesterol; (**i**) CREA: creatinine; (**j**) BUN: blood urea nitrogen; (**k**) Fe: iron; (**l**) GLU: glucose; (**m**) Ca: calcium; (**n**) P: phosphate.

The blood test on the serum biochemistry profile included a series of biomarkers such as AST, ALT, AKP and TBIL for liver functions, TG, CHOL, LDL and HDL for the lipid levels and CREA and BUN for kidney functions as well as Fe, GLU, Ca and P as other basic markers. To compare the impact of different dosage, a high and a low dose of SPION injection at 0.5 and 5 mg Fe/kg were carried out in both cirrhosis and normal groups. Prior SPION injection, AST, ALT and AKP in the cirrhosis group were significantly higher than those of the normal group, characteristic of the impaired functions due to cirrhotic damage by DDC (Fig. 2a–c). Post SPION injection, AST and ALT in the cirrhosis group increased markedly to a peak level after 24 h and then gradually decreased over 14 days (Fig. 2a,b). The high dose induced a high level of AST at 335 and ALT at 255 IU/mL while those of the low dose were at 269 and 167 IU/mL, respectively. In the normal group, the high dose of SPION injection resulted in a doubling of AST and ALT levels after 48 h whereas the low dose only led to small fluctuations of these two markers over 10 days (Fig. 2a,b). In the case of AKP, the level in the cirrhosis group increased to 379 IU/mL with the high dose after 24 h and 305 IU/mL with the low dose, but no impact of SPION was observed in the normal group (Fig. 2c). On the other hand, the levels of TBIL increased only with the high dose of SPIONs in both cirrhosis and normal group at 24 h while the low dose of SPION had no effects in either group (Fig. 2d). These results indicated that the high dose of SPION induced more significant disturbance of the liver functions within 48 h in the cirrhosis group than the normal one (the red line vs the green one, Fig. 2a,d). Moreover, within each group, the high dose of SPION resulted in much higher elevated levels of AST, ALT and TBIL within 48 h than the low dose (the red line vs the blue one and the green vs the magenta in Fig. 2a,b,d).

For the blood lipid TG level, fluctuations of TG levels were observed with high doses in both cirrhosis and normal group post SPION injection, and there were no significant differences between groups with the same dose (Fig. 2e). On the other hand, significantly high levels of CHOL, HDL and LDL were found prior to SPION injection in the cirrhosis group as compared to the normal one (Fig. 2f–h). Post the injection of the high dose, levels of these three markers in the cirrhosis group showed an initial decrease after 24 h then an increase on day 3 and then a gradual decrease to day 14. However, the low dose of SPION in the cirrhosis group mainly maintained at a plateaued level over the initial few days and then gradually decreased to the normal level. In contrast, no impact was observed post SPION injection at either dose in the normal group. In the serum markers for kidney functions, only the CREA level increased after 24 h post the high dose injection in the cirrhosis group and then returned to normal after 48 h, while none of other groups shown any significant difference (Fig. 2i,j). For the serum iron level, injection of high dose SPION produced a persistent decrease in the cirrhosis group over 14 days as the lowest (Fig. 2k). As a comparison, SPION injection in the normal mice induced a dip in the iron level that returned to normal with the low dose on day 7 and with the high dose on day 14. For other biochemistry markers, none of GLU, Ca or P exhibited any difference post SPION injection between the cirrhosis and the normal group (Fig. 2l–n).

**Figure 3:**
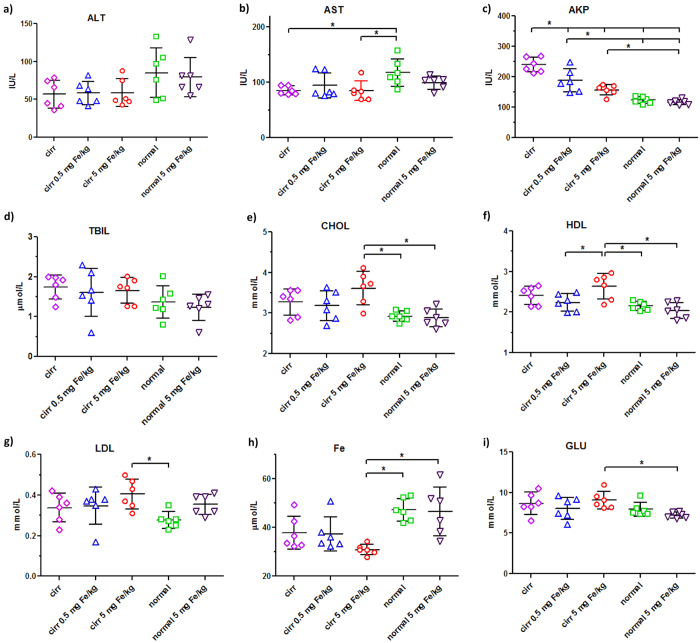
Comparison of levels of markers from serum biochemistry profiles of cirrhosis and normal mice on day 14. (**a**–**i**) selected markers from Fig. 2. Each bar graph showed the scattered plot of individual serum sample and the mean ± SD, Cirr: cirrhosis group. Statistical analysis was performed with one way ANOVA and Tukey’s multiple comparison test using the GraphPad Prism program (*P < 0.05).

All these data indicated that the injection of SPIONs at both high and low doses in the cirrhosis group resulted in a septic shock type of response to nanoparticles and such a response was only present in the normal group with the high dose within 48 h post injection. Such a shock type response of elevated serum ALT, AST and AKP has also been reported on the normal mice and rats with SPION injections^7^,^8^. A following question to these observations would be whether the septic shock response induced by nanoparticles could be recoverable over time. Therefore, levels of serum biochemistry biomarkers on day 14 post injection of both doses in the cirrhosis group and the high dose in the normal group were compared (Fig. 3). Two key references were included in the analysis to reveal the effect by SPIONs. The first one was the levels of serum biomarkers from the cirrhosis mice without SPION injection because the serum profile could be changed on day 14 due to no more feed of DDC post SPION injection. The second one was the basal levels of the normal mice without SPION injection.

Among the cirrhosis group on day 14, the impact on the levels of ALT by SPIONs was minimal with the average of cirrhosis at 57 IU/mL versus that of the normal at 85 IU/mL (Fig. 3a). Similar results were also observed in the level of AST where the level of the cirrhosis group was approximately 25 IU/mL lower than that of the normal group (Fig. 3b). On the other hand, the cirrhosis group with no SPION injection had the highest level of AKP at 240 IU/mL, followed by 188 IU/mL with 0.5 mg Fe/kg, 157 IU/mL with 5 mg Fe/kg versus 123 IU/mL of the normal groups (Fig. 3c). For the TBIL level, there was no significant difference among these five groups on day 14 (Fig. 3d). However, CHOL, HDL and LDL levels in the cirrhosis group were the highest with the 5 mg Fe/kg injection dose at 3.6, 2.6 and 0.41 mmol/L, respectively. Those of other cirrhosis groups had the average at 3.2, 2.3 and 0.34 mmol/L and those of the normal were at 2.8, 2.0 and 0.30 mmol/L (Fig. 3e–g). Similar significant difference was also observed in the iron level of the cirrhosis group with the 5 mg Fe/kg injection dose as the lowest at 31 μmol Fe/L, followed by the other two cirrhosis groups at 37 μmol Fe/L and the normal at 47 μmol Fe/L, (Fig. 3h). Other biochemistry biomarkers such as GLU were found to be no significant different between the cirrhosis and normal groups except the high injection dose in the cirrhosis group (Fig. 3i). In addition, we have also determined the iron content of the liver tissues on day 14 post SPION injection in both cirrhosis and normal groups. The iron level in the cirrhosis liver tissue with high SPION injection had an increase of 20 μg/g as compare to that of cirrhosis group with low dose or without injection (Supplementary Fig. S3). On the other hand, no difference was found between the normal groups. All these results confirmed that the cirrhosis had characteristic decreased serum levels of ALT, AST and Fe and increased levels of AKP, CHOL, HDL and LDL as compared to the normal one. More importantly, it was revealed that the high dose of SPION injection in the cirrhosis group resulted in irrecoverable changes of CHOL, HDL, LDL and iron levels after 14 days whereas the impact of a 0.5 mg Fe/kg dose was minimal in the cirrhosis group. In addition, our results indicated that the high dose of SPION injection in the normal group produced septic-shock responses that were recoverable within 14 days. Overall, it was implied that a high dose of SPION injection at 5 mg Fe/kg in the cirrhosis group could potentially be a serious safety concern due to the aggravated burden on cirrhosis by the acute iron overload.

### SPIONs induced distinct expression changes of genes of toxicity pathways in cirrhosis group

**Figure 4:**
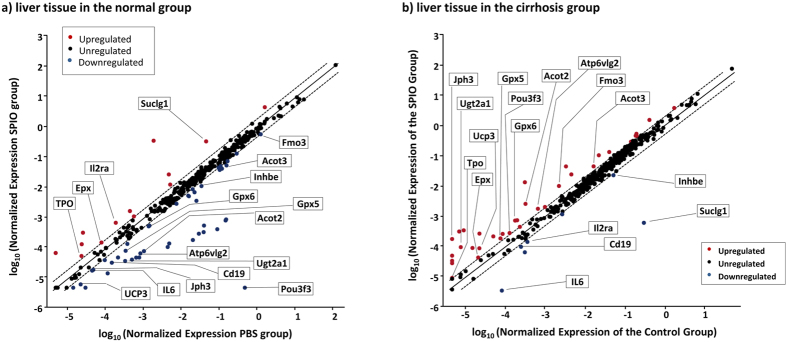
Correlation scatter plots of the expression levels of 370 genes among more than thirteen pathways in the liver and spleen tissues of the normal and cirrhosis group post the SPION injection versus the controls by RT^2^ PCR array analysis. The gene levels of each sample were first normalized with four internal housekeeping genes and then plotted according to the expression levels in the SPION group versus those of the control. Genes with more than 2-folds regulation were considered significant with the red dots for the upregulated and the blue ones for the down-regulated. The dash lines indicated the range of 2-folds changes. The data presented were from the results of RT^2^ PCR array analysis of two independent tissue samples in each group.

The impact post the high dose of 5 mg Fe/kg SPION injection in the cirrhosis group might possibly be due to different responses in the toxicity pathways in the cirrhotic liver from those in the normal group. Thus, the acute responses to SPIONs at mRNA levels in the molecular toxicity pathways were investigated at 24 h post injection by a high throughput real time PCR array analysis. The molecular toxicity PCR array contained a pool of 370 genes from pathways of immunotoxicity, oxidative stress and antioxidant response, mitochondrial energy metabolism, DNA damage and repair, apoptosis, necrosis, CYP450s and phase I drug metabolism, fatty acid metabolism, phospholipidosis, steatosis, cholestatis, heatshock response and endothelial reticulum (ER) stress and unfolded protein response^27^. The mRNA expressions of these genes in each tissue group were assessed, normalized and then plotted in the scatter plot to show both expression levels and changes post SPION injection (Fig. 4). While most of genes were close to the diagonal line as not being significantly impacted (Fig. 4), there were multiple spots outside the diagonal dashed lines of 2-folds changes that were considered to be significantly impacted^21^,^22^. In both groups studied, there were a total of eighty-two genes being regulated post SPION injection (Supplementary Table S1). In the normal liver, there were thirteen upregulated and forty-four downregulated genes post SPION injection (red vs blue dots, Fig. 4a). In contrast, there were thirty-five upregulated genes and only seven down-regulated genes in the cirrhotic liver post SPION injection (Fig. 4b). Among these up- and down-regulated genes, there were seventeen genes found present in both groups as labeled in Fig. 4. However, most of them differed dramatically in the cirrhosis group from those of the normal one except three genes Tpo, Epx and Inhbe, indicating a disparate pattern of responses in the molecular toxicity to SPIONs in the cirrhosis.

Genes with expression by more than 2-folds changes were categorized into the 13 major molecular toxicity pathways as listed in Supplementary Table S1. First, the immunotoxicity was a major contributor to the molecular toxicity responses post SPION injection in the cirrhotic liver. For examples, genes from inflammatory responses such as colony-stimulating factor (Csf2), IL-4, IL-2 and IL-5 were dominantly elevated in the cirrhotic liver. In addition, several genes involved in both immunotoxicity and lipid metabolism pathway such as Cyp4a14 and panaxonase 1 (Pon1) were also upregulated in the cirrhotic liver post SPION injection^28^,^29^,^30^. In contrast, SPION injection in the normal liver mainly induced downregulation of genes including Pou3f3, CD4 and IL-10. Secondly, the oxidative stress induced by iron overload was more pronouncedly observed in the cirrhotic liver with the significantly upregulated glutathione peroxidases Gpx5 and Gpx6 and Ucp3 that were otherwise downregulated in the normal liver^31^. Thirdly, in the mitochondrial energy metabolism and DNA damage and repair pathways, only three out of 59 genes assessed were upregulated post SPION injection in the cirrhotic liver. However, one gene Suclg1 was considerably downregulated in the cirrhosis versus up-regulation in the normal group. In the cell death pathways, the TNF-induced necrotic pathway was clearly elevated in the cirrhotic liver post SPION injection via increased expression of junctopilin 3 (Jph3), lysomal H^+^ transporting ATPase (Atp6v1g2) and TNF (Fasl) and decreased level of defensin β_6_ (Defb1)^32^. However, downregulation of genes in the apoptotic pathway such as Casp8 and Atp6v1g2 was observed in the normal liver post SPION injection. In the category of drug metabolism, three genes including CYP1a2, CYP2e1 and Fmo3 were upregulated in the cirrhotic liver post SPION injection as compared with upregulated CYP1a1 and downregulated Fmo3 in the normal liver. Increased expression of CYP 2e1 has been reported to be involved in the elevated lipid metabolism in clinical patients^33^. Consistently, nine genes in the fatty acid metabolism, phospholipidosis and steatosis pathways including Acot2, Ugt2a1, Cd36 and Retn were upregulated in the cirrhotic liver post SPION injection accompanied with elevated Tgfb1 and sodium bile acid cotransporter (Slc10a1) of the cholestasis pathway^28^. On the contrary, only three genes of the lipid metabolism were upregulated in the normal liver post SPION injection (Supplementary Table S1), whereas sixteen genes were downregulated implying an overall downregulation of lipid metabolism in the normal group. Finally, the cirrhotic liver had two upregulated genes in the ER stress and unfolded protein response while the normal one had five downregulated and two upregulated ones post SPION injection (Supplementary Table S1). Clearly, the high dose of SPION injection at 5 mg Fe/kg induced significant and disparate changes in the toxicity pathways in the cirrhosis liver as compared to the normal one.

## Discussion

**Figure 5:**
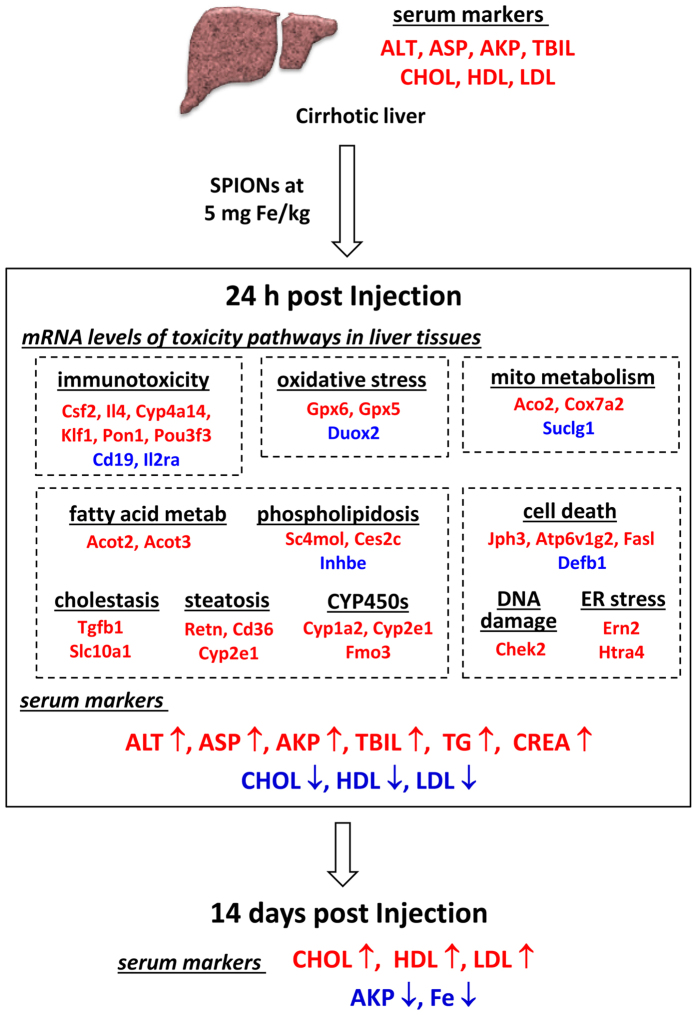
Summary of toxicity assessment of high dose of SPION injection in the cirrhosis group. Only genes with expression levels reasonably detected (Ct < 30) by RT^2^ PCR from Supplementary Table S2 of the cirrhosis group were selected whereas the genes with relatively low expression were excluded (Ct > 30). The down-regulated genes or decreased serum biomarkers are shown in the blue color whereas the upregulated ones are in red.

The overall safety assessment of the high dose of SPIONs in the cirrhosis group was obtained by the combination of both regulated genes of the molecular toxicity pathways and serum biochemistry profiles as shown in Fig. 5. Only genes had significant expression levels were considered in the overall assessment. Prior injection, the cirrhosis model had abnormal serum levels of liver function markers and cholesterols. Upon injection of SPIONs at 5 mg Fe/kg, significant expression changes of a distinct set of genes in the immunotoxicity, oxidative stress and mitochondrial metabolism, lipid metabolism and cell death related pathways were induced in the liver tissue at 24 h. This concurred with a septic shock type of responses in the blood serum with elevated levels of liver and kidney function markers, TG and at same time, decreased cholesterol levels. The acute phase responses then resulted in elevated serum cholesterol levels, persistent low serum iron level, decreased elevated level of AKP and high iron content in the liver tissue over 14 days. Since most of iron from the SPIONs was retained in the cirrhotic liver at 24 h post injection (Table 1), these toxicity consequences by SPIONs were consistent with responses to enhanced oxidative stress by the iron overload^8^,^10^,^11^. It has been showed that in the normal rat, injection of an oleic acid-pluronic-coated SPION at 10 mg Fe/kg induced the shock type responses of elevated serum ALT, AST and AKP levels and the production of lipid hydroperoxide in the liver tissue^8^. The interactions of genes of toxicity pathways could thus be hypothesized that the increased lipid peroxidation with excess iron in the cirrhotic liver lead to responses with elevated lipid metabolism including fatty acid metabolism, phospholipidosis, steatosis and cholestasis^33^. Increased mitochondrial energy metabolism and consequent DNA oxidative damage might contribute additional oxidative stress, which trigged the activation of cell death pathway via the TNF-induced necrosis^32^. In addition, the inflammatory responses in the necroinflammatory environment contributed further immunotoxicity post SPION injection in cirrhotic liver. As a result, impaired liver functions occurred and cholesterols were depleted at 24 h post injection (Fig. 2). More importantly, although most of the liver functions were recovered over time, AKP level decreased significantly post SPION injection on day 14 with high dose SPION injection in the cirrhosis group versus that of cirrhosis control without SPIONs (Fig. 3). Considering that the serum profile of cirrhosis included low levels of ALT and AST and elevated AKP, the decreased elevated AKP level post high dose SPION injection might possibly be an indication of decreased liver function as a consequence of upregulated cell-death responses observed in the toxicity pathways. In addition, elevated serum cholesterol levels, suppressed serum iron level and high iron content in the liver remained persistently after 14 days as compared to the normal one, indicating disruption of homeostasis in cirrhosis liver by iron overload. It has been shown that hepatic iron level played a key part in fibrosis of non-alcoholic fatty liver disease and correlated to the progression of steatosis to steatohepatitis^15^,^31^,^32^. In contrast, the impact by the high dose of SPION injection were recoverable within 14 days in the normal group, which was consistent with the reported low cytotoxicity of our biocompatible SPIONs in the liver cells below 500 μg/mL^23^ and other reported SPIONs^6^. Clearly, the iron overload has dramatic different impact with cirrhosis in the liver. Thus, considering that the injected dose at 5 mg Fe/kg in mice was equivalent to 0.4 mg Fe/kg for human based on the body surface area^25^, the impacts by the SPIONs would present a potential safety issue and a high risk factor in the cirrhosis.

In conclusion, we demonstrated that injection of SPION solution at 5 mg Fe/kg in mice had potential safety concern in cirrhosis due to the iron overload in the liver. The manifestations of short-term impaired liver functions, decreased cholesterols and significantly altered gene expressions of toxicity pathways as well as extended elevated cholesterol levels, persistent deficient iron level in blood and decreased liver function implied that aggravated burden might occur in patients with cirrhosis when such a dose of SPIONs were used. In contrast, the same SPION dosage in the normal group is not a safety concern due to the restoration of liver functions over time. However, the normal group usually does not need the application of SPIONs as a contrast agent to improve diagnosis in MRI. On the other hand, our study also indicated that with a low dose of SPIONs at 0.5 mg Fe/kg in mice, there was no significant difference among the serum markers with or without injection after 14 days and the short-term septic shock type of responses were minimal (Fig. 2). In addition, such a low dose has been demonstrated effective to enhance the detection of liver tumors in the MRI imaging diagnosis. Moreover, this optimal dose of the synthesized SPION nanoclusters may be unique to the biocompatible poly-*L*-lysine and hydroxyethyl starch coating materials because different compositions of protein corona had been found on the SPION with different coating materials and/or charges that could consequently have different level of iron overload in the liver^34^,^35^. Therefore, future applications of SPIONs in different cancer patients should be optimized with different dosages to accommodate their specific needs due to various clinical dysfunctions.

## Methods

### Ethics Statement

The animal protocol was approved by the Animal Care and Use Committee of College of Life Science and Technology at Huazhong University of Science and Technology. All experiments were performed in accordance with the ethical principles of relevant guidelines and regulations.

### Cirrhosis mouse model

SFP male BALB/c mice (approximately 20 g) were obtained from Hubei Provincial Laboratory Animal Center, China. The cirrhotic liver model was established according to the reported procedures^24^. Briefly, a suspension of diethyl 1,4-dihydro-2,4,6-trimethyl-3,5-pyridine-dicarboxylate (DDC, Acros Organics, NJ, USA) in corn oil (10 mg/mL; pharmaceutical grade, Aladdin, Shanghai, China) was fed to mice by oral gavage at a dose of 0.25 mg/g body weight twice a week continuously for four weeks. The lesions of cirrhosis in the liver were generally formed at week six and confirmed by histopathology with Sirius red staining.

### Iron levels in the liver and spleen post SPION injection

The biocompatible SPION nanocluster solution containing 3.5 μg/mL poly*-L*-lysine in water was prepared as reported previously^23^. The hydrodynamic size and zeta-potential of the SPION were determined in water with a Nano-ZS90 particle analyzer (Malvern, United Kingdom). The transmission electron microscopic analysis of the synthesized SPIONs was carried out with a Tecnai G2 20 spectrometer (FEI Incorporated, USA) using phosphotungstic acid staining. The stability of SPIONs at a concentration of 2 mg Fe/mL in PBS solution containing 10% fetal bovine serum at 37 °C over 7 days was determined by measuring the hydrodynamic size of sequential aliquots of solution.

The synthesized SPION solution was intravenously injected in the cirrhosis mice (in week 6) at a single dose of 5 mg Fe/kg body weight. A group of cirrhosis mice (four mice per group) without the SPION injection was included as the control. As a comparison, groups of normal naïve mice with and without SPION injection were also included. The liver and spleen tissues were collected at 24 h post iv injection for the iron assessment and pathology evaluation. For the iron level determination, liver and spleen tissues were weighted and digested with perchloric acid/nitric acid (1:8) at 320 °C for 20 min. The iron concentration was determined by an inductively coupled plasma atomic absorbance spectrometer (SpectrAA-4, Varian, USA). Statistical analysis was performed with one way ANOVA and Tukey’s multiple comparison test using the GraphPad Prism program (GraphPad Software, USA).

### Serum biochemistry profiles of cirrhosis mice post SPION injection

A single dose of prepared SPION solution in water at 0.5 or 5 mg Fe/kg body weight (approximately 100 μL) was intravenously injected in the mice at the end of cirrhosis model development in week six. Blood from the mice was collected on day 1, 2, 3, 5, 7, 9 and 14 post SPION injection (six mice per time point of each group). The blood test for the serum biochemistry profile included levels of alanine aminotransferase (ALT), aspartate aminotransferase (AST), alkaline phosphatase (AKP), total bilirubin (TBL), glucose (GLU), iron (Fe), cholesterol (CHOL), high-density lipoprotein cholesterol (HDL), low density lipoprotein cholesterol (LDL), triglycerides (TG), creatinine (CREA), blood urea nitrogen (BUN), calcium (Ca) and phosphate (P). As a comparison, the serum biochemistry profiling of the normal mice post SPION injection at the same doses was also carried out. In addition, levels of serum biochemistry markers in the cirrhosis and normal mice without SPION injection were obtained at the end of profiling study (day 14) and used as the reference levels. All the data obtained were analyzed by GraphPad Prism program with one way ANOVA and Tukey’s multiple comparison statistical test.

### RT^2^ PCR array analysis of the cirrhosis and normal liver samples

Samples from the liver tissues obtained at 24 h post iv injection were snap frozen in liquid nitrogen and stored at −80 °C until use. The total RNA was isolated from tissues with the RNeasy microarray tissue kit (Qiagen, CA, USA) according to the manufacturer’s instructions. The cDNA synthesis and the real time PCR was carried out with RT^2^ First strand kit (Qiagen) and the 384-well molecular toxicology pathwayfinder array (PAMM-3401Z, Qiagen) in the 7900HT fast real-time PCR system (Applied Biosystems, CA, USA). The names and descriptions of 370 genes of the toxicity pathways were listed in the Supplementary Table S2 and can also be found online (PAMM-3401Z, Qiagen). The cycling program and threshold cycle (Ct) values were determined according to the manufacturer’s protocol. The resulting data were uploaded and analyzed with the web-based PCR array data analysis software ( www.SABiosciences.com/pcrarraydataanalysis.php). Briefly, the Ct cut-off value for the data loaded was set to 35, and the Ct values for all wells were then normalized by those of housekeeping genes including β-actin (Actb), β_2_ microglobulin (B2m), glyceraldehyde 3-phosphate (Gadph) and heatshock protein 90β (Hsp90ab1). The folds of changes and the scatter plots of each paired groups (the SPION injection group vs the no SPION injection group) were obtained from the online website analysis. The data presented in the tables and figures were from the results of RT^2^ PCR array analysis of two independent tissue samples of each group.

## Additional Information

**How to cite this article**: Wei, Y. *et al*. Iron overload by Superparamagnetic Iron Oxide Nanoparticles is a High Risk Factor in Cirrhosis by a Systems Toxicology Assessment. *Sci. Rep.* **6**, 29110; doi: 10.1038/srep29110 (2016).

## Supplementary Material

Supplementary Information

## Figures and Tables

**Table 1:** The amount of excess iron found in the whole liver and spleen at 24 h post SPION injection (5 mg Fe/kg).

	**Cirrhotic**		**Normal**	
	**Liver**	**Spleen**	**Liver**	**Spleen**
weight (g)^1^	1.636 ± 0.380*	0.109 ± 0.014	0.842 ± 0.097	0.110 ± 0.009
excess Fe (μg) in the organ^2^	100.4 ± 23.3	14.9 ± 1.9	114.8 ± 13.3	2.8 ± 0.2
% of excess Fe in SPIONs injected^3^	80.3 ± 18.2	11.9 ± 1.5	91.8 ± 10.6	2.2 ± 0.2

^1^The weights of the liver and the spleen were the mean ± SD obtained from eight mice in the cirrhosis or control group. There was no statistical difference between the mice injected with SPIONs and the control without SPION injection.

^2^The excess Fe found in the organ was calculated from the weight of the organ times the difference of Fe levels between the mice injected with SPIONs and the controls from Fig. 1.

^3^The percent of excess Fe in SPIONs injected was obtained by the amount of excess Fe found in the organ divided by the total iron (125 μg) in the SPIONs injected.

^*^P < 0.01 as compared with that of the normal liver.
