# Supplementary material for: Iron overload by Superparamagnetic Iron Oxide Nanoparticles is a High Risk Factor in Cirrhosis by a Systems Toxicology Assessment
Source: Sci Rep. 2016 Jun 30;6:29110. doi: 10.1038/srep29110 (PMC4928111; doi:10.1038/srep29110)
Supplement: Supplementary Information [file srep29110-s1.pdf]

Iron overload by Superparamagnetic Iron Oxide Nanoparticles is a High Risk Factor in Cirrhosis by a Systems Toxicology Assessment

Yushuang Wei[†], Mengzhu Zhao[†], Fang Yang[†], Yang Mao[†], Hang Xie[‡], Qibing Zhou[†,]*

[†]Department of Nanomedicine & Biopharmaceuticals, National Engineering Research Center for Nanomedicine, Huazhong University of Science and Technology, Wuhan, Hubei, China

[‡]Division of Viral Products, Office of Vaccines Research and Review, Center for Biologics Evaluation and Research, United States Food and Drug Administration, Silver Spring, Maryland, United States of America

Supplementary Materials

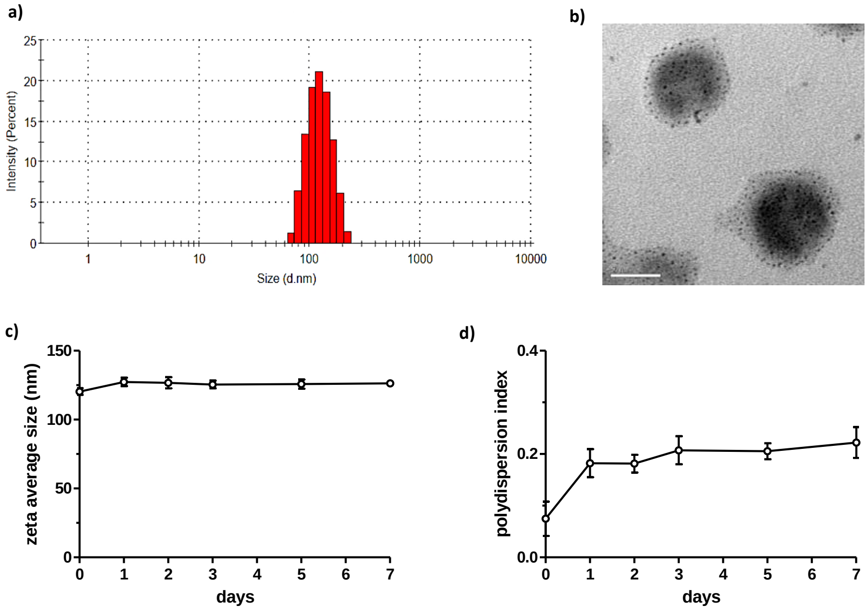

Figure S1. Physiological properties of SPIONs and their stability over time. a) the size distribution of SPIONs in water as measured by dynamic light scattering; b) TEM image of synthesized SPIONs (the scale bar at the bottom was 50 nm); c) and d) the stability of SPIONs at 37 °C over 7 days in the PBS solution containing 10% fetal bovine serum; the data in the graphs were presented as the average of the triplicated measurements with the standard deviation as the error bar.

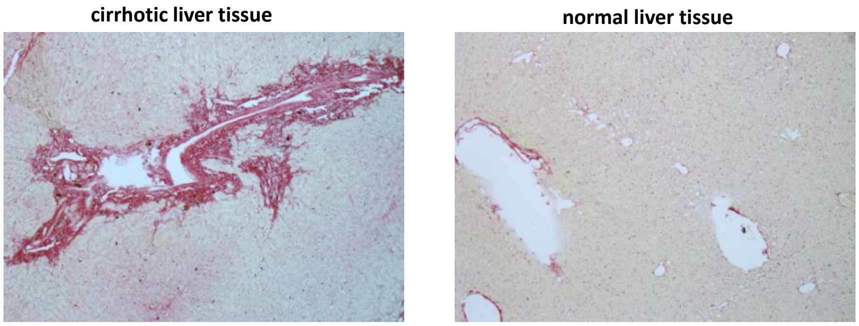

Figure S2. Pathology analysis of the liver tissues by Sirius red staining in the cirrhosis model as compared to the normal one. The liver tissues were obtained in week six post oral gavage of a suspension of DDC in corn oil for four weeks. The scar tissue was stained positively as the dark red color as shown in the left top panel.

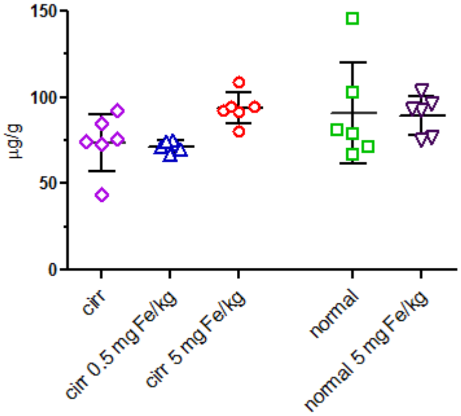

Figure S3. Iron level of the liver tissues in the cirrhosis and normal groups on day 14 post the SPION injection. Cirr: cirrhosis group without SPION injection; cirr 0.5 mg Fe/kg: cirrhosis group with SPION injection at 0.5 mg Fe/kg; cirr 5mg Fe/kg: cirrhosis group with SPION injection at 5 mg Fe/kg; normal: normal group without SPION injection; normal 5 mg Fe/kg: normal group with SPION injection at 0.5 mg Fe/kg. Each subgroup had six mice and statistical analysis indicated there was no significant difference.

Supplementary Table S1. Regulation of gene expression of the molecular toxicity pathways in the liver tissues by more than 2-folds changes at 24 h post SPION injection (5 mg Fe/kg).[1]

gene symbol	Description	normal liver	cirrhotic liver	pathways involved
Cd4	CD4 antigen	**-12.75**[A]	–*	Immunotoxicity
Cd19	CD19 antigen	**-4.27**[B]	**-2.44**[A]	Immunotoxicity
Csf2	Colony stimulating factor 2 (granulocyte-macrophage)	–	**13.81**	Immunotoxicity
Cyp4a14	Cytochrome P450 4a14	–	**3.36**	Immunotoxicity
Ifng	Interferon gamma	**-2.28**[B]	–	Immunotoxicity
Il2	Interleukin 2	–	**11.43**[B]	Immunotoxicity, Cholestasis
Il2ra	Interleukin 2 receptor, alpha chain	**3.89**	**-2.44**	Immunotoxicity
Il4	Interleukin 4	–	**12.52**[A]	Immunotoxicity
Il5	Interleukin 5	–	**6.29**[B]	Immunotoxicity
Il6	Interleukin 6	**-2.37**[B]	**-22.72**[B]	Immunotoxicity, Cholestasis
Il10	Interleukin 10	**-7.47**[B]	–	Immunotoxicity, Cholestasis
Klf1	Kruppel-like factor 1 (erythroid)	–	**3.18**[A]	Immunotoxicity
Pon1	Paraoxonase 1	–	**2.77**	Immunotoxicity
Pou3f3	POU domain, class 3, transcription factor 3	**-94008**[A]	**2.27**[A]	Immunotoxicity
Aass	Aminoadipate-semialdehyde synthase	**-164.22**	–	Oxidative Stress & Response
Duox2	Dual oxidase 2	–	**-4.66**[A]	Oxidative Stress & Response
Epx	Eosinophil peroxidase	**2.05**[B]	**2.17**[B]	Oxidative Stress & Response
Gpx5	Glutathione peroxidase 5	**-3.98**[A]	**2.57**[A]	Oxidative Stress & Response
Gpx6	Glutathione peroxidase 6	**-2.50**	**3.88**	Oxidative Stress & Response
Tpo	Thyroid peroxidase	**2.47**[B]	**2.09**[B]	Oxidative Stress & Response
Txnip	Thioredoxin interacting protein	**-2.52**	–	Oxidative Stress & Response
Ucp3	Uncoupling protein 3 (mitochondrial, proton carrier)	**-3.36**[B]	**4.12**[B]	Oxidative Stress & Response, Mitochondria Energy Metabolism
Aco2	Aconitase 2, mitochondrial	–	**5.49**	Mitochondria Energy Metabolism
Cox7a2	Cytochrome c oxidase, subunit VIIa 2	–	**4.53**[A]	Mitochondria Energy Metabolism
Ogdhl	Oxoglutarate dehydrogenase-like	**14.94**[B]	–	Mitochondria Energy Metabolism
Suclg1	Succinate-CoA ligase, GDP-forming, alpha subunit	**8.01**	**-498.17**	Mitochondria Energy Metabolism
Atm	Ataxia telangiectasia mutated homolog (human)	**-11.44**[A]	–	DNA Damage & Repair
Cdkn1a	Cyclin-dependent kinase inhibitor 1A (P21)	**6.16**	–	DNA Damage & Repair
Chek2	CHK2 checkpoint homolog (*S. pombe*)	–	**2.12**	DNA Damage & Repair
Ercc3	Excision repair cross-complementing rodent repair deficiency, complementation group 3	**-2.90**	–	DNA Damage & Repair
Gadd45a	Growth arrest and DNA-damage-inducible 45 alpha	**-6.49**	–	DNA Damage & Repair, Apoptosis, ER Stress & Unfolded Protein Response
Casp8	Caspase 8	**-110.32**	–	Apoptosis
Cd40	CD40 antigen	**-2.61**[A]	–	Apoptosis
Cd40lg	CD40 ligand	**-4.18**[A]	–	Apoptosis
Cflar	CASP8 and FADD-like apoptosis regulator	**-2.01**	–	Apoptosis
Fasl	Fas ligand (TNF superfamily, member 6)	–	**4.25**	Apoptosis
Tnfrsf10b	Tumor necrosis factor receptor superfamily, member 10b	**-2.49**	–	Apoptosis
Atp6v1g2	ATPase, H+ transporting, lysosomal V1 subunit G2	**-11.90**[A]	**2.79**	Necrosis
Defb1	Defensin beta 1	–	**-2.23**	Necrosis
Dpysl4	Dihydropyrimidinase-like 4	**5.99**[B]	–	Necrosis
Galnt5	UDP-N-acetyl-alpha-D-galactosamine:polypeptide N-acetylgalactosaminyltransferase 5	–	**7.71**[B]	Necrosis
Jph3	Junctophilin 3	**-2.38**[B]	**40.77**[A]	Necrosis
Olfr1404	Olfactory receptor 1404	–	**6.43**[B]	Necrosis
S100a7a	S100 calcium binding protein A7A	**-5.81**[B]	–	Necrosis
Cyp1a1	Cytochrome P450 1a1	**2.09**	–	CYP450s & Drug Metabolism, Immunotoxicity
Cyp1a2	Cytochrome P450 1a2	–	**2.33**	CYP450s & Drug Metabolism
Cyp2e1	Cytochrome P450 2e1	–	**2.20**	CYP450s & Drug Metabolism, Steatosis
Fmo3	Flavin containing monooxygenase 3	**-2.01**	**4.67**	CYP450s & Drug Metabolism

Acot2	Acyl-CoA thioesterase 2	-12.48[A]	8.33	Fatty Acid Metabolism
Acot3	Acyl-CoA thioesterase 3	-2.72	2.78	Fatty Acid Metabolism
Acot7	Acyl-CoA thioesterase 7	-2.67	–	Fatty Acid Metabolism
Acot8	Acyl-CoA thioesterase 8	-104.98	–	Fatty Acid Metabolism
Acot9	Acyl-CoA thioesterase 9	-38.51[A]	–	Fatty Acid Metabolism
Acot12	Acyl-CoA thioesterase 12	-2.09	–	Fatty Acid Metabolism
Acox2	Acyl-Coenzyme A oxidase 2, branched chain	-2.64	–	Fatty Acid Metabolism
Echs1	Enoyl Coenzyme A hydratase, short chain, 1, mitochondrial	204.58	–	Fatty Acid Metabolism
Ces2c	Carboxylesterase 2C	–	2.50	Phospholipidosis
Inhbe	Inhibin beta E	-2.87	-2.24	Phospholipidosis
Sc4mol	Sterol-C4-methyl oxidase-like	–	3.76	Phospholipidosis
Ugt2a1	UDP glucuronosyltransferase 2A1	-8.40[A]	13.20[B]	Phospholipidosis
Abcb1a	ATP-binding cassette, B1A (MDR/TAP)	-3.01	–	Cholestasis
Abcb4	ATP-binding cassette, B4 (MDR/TAP)	-202.81	–	Cholestasis
Abcc1	ATP-binding cassette, C1 (CFTR/MRP)	-15.17[A]	–	Cholestasis
Abcc3	ATP-binding cassette, C3 (CFTR/MRP)	-2.03	–	Cholestasis
Cyp7a1	Cytochrome P450 7a1	-2.04	–	Cholestasis
Slc10a1	Solute carrier family 10 (sodium/bile acid cotransporter family), member 1	–	2.08	Cholestasis
Tgfb1	Transforming growth factor, beta 1	–	45.18	Cholestasis
Aqp4	Aquaporin 4	-2.42	–	Steatosis
Cd36	CD36 antigen	–	4.79	Steatosis
Pnpla3	Patatin-like phospholipase domain containing 3	2.63	–	Steatosis
Retn	Resistin	–	47.91[A]	Steatosis
Scd1	Stearoyl-Coenzyme A desaturase 1	3.06	–	Steatosis
Syt1	Synaptotagmin I	-2.14	–	Steatosis
Cryaa	Crystallin, alpha A	-2.75[B]	–	Heat Shock Response
Cryab	Crystallin, alpha B	-32.46[A]	–	Heat Shock Response
Dnaja2	DnaJ (Hsp40) homolog, subfamily A, member 2	-171.73	–	Heat Shock Response
Hspa1a	Heat shock protein 1A	4.03	–	Heat Shock Response
Hspb7	Heat shock protein family, member 7 (cardiovascular)	13.37[A]	–	Heat Shock Response
Atf4	Activating transcription factor 4	-68.31	–	ER Stress & Unfolded Protein Response
Atf6	Activating transcription factor 6	-93.12	–	ER Stress & Unfolded Protein Response
Ern2	Endoplasmic reticulum (ER) to nucleus signalling 2	–	40.17[A]	ER Stress & Unfolded Protein Response
Htra4	HtrA serine peptidase 4	–	2.01	ER Stress & Unfolded Protein Response

[1]Only genes from RT^2 PCR analysis by more than 2-folds changes were presented in the table with the red color for the upregulated and the blue for the downregulated. The folds of upregulated were calculated as the levels of expression in the SPION group over those of the control group without SPION injection whereas the downregulated ones were the negative inverse of the levels of expression in the SPION group over those of the control group.

[*]The change of expression was less than 2-folds.

[A]The gene expression level was reasonably detected in one group yet relatively low in the other group (Ct>30), possibly due to the variations between the samples.

[B]The gene expression levels were low in the both groups (Ct>30), thus the folds of changes could be false positive, possibly due to large variations between the samples.

Table S2. Symbols and description of the 370 genes determined in the 384-well molecular toxicology pathwayfinder array (PAMM-3401Z, Qiagen).

No	Symbol	Description	Refseq
1	9430015G10Rik	RIKEN cDNA 9430015G10 gene	NM_145557
2	Aass	Aminoadipate-semialdehyde synthase	NM_013930
3	Abcb1a	ATP-binding cassette, sub-family B (MDR/TAP), member 1A	NM_011076
4	Abcb1b	ATP-binding cassette, sub-family B (MDR/TAP), member 1B	NM_011075
5	Abcb4	ATP-binding cassette, sub-family B (MDR/TAP), member 4	NM_008830
6	Abcc1	ATP-binding cassette, sub-family C (CFTR/MRP), member 1	NM_008576
7	Abcc2	ATP-binding cassette, sub-family C (CFTR/MRP), member 2	NM_013806
8	Abcc3	ATP-binding cassette, sub-family C (CFTR/MRP), member 3	NM_029600
9	Abl1	C-abl oncogene 1, non-receptor tyrosine kinase	NM_009594
10	Acaa1a	Acetyl-Coenzyme A acyltransferase 1A	NM_130864
11	Acaa2	Acetyl-Coenzyme A acyltransferase 2 (mitochondrial 3-oxoacyl-Coenzyme A thiolase)	NM_177470
12	Acaca	Acetyl-Coenzyme A carboxylase alpha	NM_133360
13	Acad11	Acyl-Coenzyme A dehydrogenase family, member 11	NM_175324
14	Acad9	Acyl-Coenzyme A dehydrogenase family, member 9	NM_172678
15	Acadl	Acyl-Coenzyme A dehydrogenase, long-chain	NM_007381
16	Acadm	Acyl-Coenzyme A dehydrogenase, medium chain	NM_007382
17	Acads	Acyl-Coenzyme A dehydrogenase, short chain	NM_007383
18	Acadsb	Acyl-Coenzyme A dehydrogenase, short/branched chain	NM_025826
19	Acadvl	Acyl-Coenzyme A dehydrogenase, very long chain	NM_017366
20	Acat1	Acetyl-Coenzyme A acetyltransferase 1	NM_144784
21	Acat2	Acetyl-Coenzyme A acetyltransferase 2	NM_009338
22	Acly	ATP citrate lyase	NM_134037
23	Aco1	Aconitase 1	NM_007386
24	Aco2	Aconitase 2, mitochondrial	NM_080633
25	Acot12	Acyl-CoA thioesterase 12	NM_028790
26	Acot2	Acyl-CoA thioesterase 2	NM_134188
27	Acot3	Acyl-CoA thioesterase 3	NM_134246
28	Acot6	Acyl-CoA thioesterase 6	NM_172580
29	Acot7	Acyl-CoA thioesterase 7	NM_133348
30	Acot8	Acyl-CoA thioesterase 8	NM_133240
31	Acot9	Acyl-CoA thioesterase 9	NM_019736
32	Acox1	Acyl-Coenzyme A oxidase 1, palmitoyl	NM_015729
33	Acox2	Acyl-Coenzyme A oxidase 2, branched chain	NM_053115
34	Acox3	Acyl-Coenzyme A oxidase 3, pristanoyl	NM_030721
35	Adh1	Alcohol dehydrogenase 1 (class I)	NM_007409
36	Adk	Adenosine kinase	NM_134079
37	Ahr	Aryl-hydrocarbon receptor	NM_013464
38	Ahsg	Alpha-2-HS-glycoprotein	NM_013465
39	Akt1	Thymoma viral proto-oncogene 1	NM_009652
40	Alb	Albumin	NM_009654
41	Aldh1a1	Aldehyde dehydrogenase family 1, subfamily A1	NM_013467
42	Aldh2	Aldehyde dehydrogenase 2, mitochondrial	NM_009656
43	Amfr	Autocrine motility factor receptor	NM_011787
44	Apaf1	Apoptotic peptidase activating factor 1	NM_009684

45	Apex1	Apurinic/apyrimidinic endonuclease 1	NM_009687
46	Apoa5	Apolipoprotein A-V	NM_080434
47	Apoe	Apolipoprotein E	NM_009696
48	Apof	Apolipoprotein F	NM_133997
49	Aqp4	Aquaporin 4	NM_009700
50	Asah1	N-acylsphingosine amidohydrolase 1	NM_019734
51	Asns	Asparagine synthetase	NM_012055
52	Atf4	Activating transcription factor 4	NM_009716
53	Atf6	Activating transcription factor 6	NM_001081304
54	Atm	Ataxia telangiectasia mutated homolog (human)	NM_007499
55	Atp6v1g2	ATPase, H+ transporting, lysosomal V1 subunit G2	NM_023179
56	Atp8b1	ATPase, class I, type 8B, member 1	NM_001001488
57	Bad	BCL2-associated agonist of cell death	NM_007522
58	Bak1	BCL2-antagonist/killer 1	NM_007523
59	Bax	Bcl2-associated X protein	NM_007527
60	Bcl2	B-cell leukemia/lymphoma 2	NM_009741
61	Bcl2l1	Bcl2-like 1	NM_009743
62	Bcl2l11	BCL2-like 11 (apoptosis facilitator)	NM_009754
63	Bid	BH3 interacting domain death agonist	NM_007544
64	Birc3	Baculoviral IAP repeat-containing 3	NM_007464
65	Bmf	Bcl2 modifying factor	NM_138313
66	Brca1	Breast cancer 1	NM_009764
67	Brca2	Breast cancer 2	NM_009765
68	C3	Complement component 3	NM_009778
69	C9	Complement component 9	NM_013485
70	Casp1	Caspase 1	NM_009807
71	Casp2	Caspase 2	NM_007610
72	Casp3	Caspase 3	NM_009810
73	Casp7	Caspase 7	NM_007611
74	Casp8	Caspase 8	NM_009812
75	Casp9	Caspase 9	NM_015733
76	Cat	Catalase	NM_009804
77	Cd19	CD19 antigen	NM_009844
78	Cd36	CD36 antigen	NM_007643
79	Cd4	CD4 antigen	NM_013488
80	Cd40	CD40 antigen	NM_011611
81	Cd40lg	CD40 ligand	NM_011616
82	Cd44	CD44 antigen	NM_009851
83	Cd80	CD80 antigen	NM_009855
84	Cd86	CD86 antigen	NM_019388
85	Cd8a	CD8 antigen, alpha chain	NM_001081110
86	Cdkn1a	Cyclin-dependent kinase inhibitor 1A (P21)	NM_007669
87	Ces2c	Carboxylesterase 2C	NM_145603
88	Cflar	CASP8 and FADD-like apoptosis regulator	NM_009805
89	Chek1	Checkpoint kinase 1 homolog (S. pombe)	NM_007691
90	Chek2	CHK2 checkpoint homolog (S. pombe)	NM_016681
91	Commd4	COMM domain containing 4	NM_025417
92	Comt	Catechol-O-methyltransferase	NM_007744

93	Cox6b1	Cytochrome c oxidase, subunit VIb polypeptide 1	NM_025628
94	Cox7a2	Cytochrome c oxidase, subunit VIIa 2	NM_009945
95	Cox8a	Cytochrome c oxidase, subunit VIIIa	NM_007750
96	Cpt1a	Carnitine palmitoyltransferase 1a, liver	NM_013495
97	Cpt1b	Carnitine palmitoyltransferase 1b, muscle	NM_009948
98	Cpt2	Carnitine palmitoyltransferase 2	NM_009949
99	Crat	Carnitine acetyltransferase	NM_007760
100	Crot	Carnitine O-octanoyltransferase	NM_023733
101	Cryaa	Crystallin, alpha A	NM_013501
102	Cryab	Crystallin, alpha B	NM_009964
103	Cs	Citrate synthase	NM_026444
104	Csf2	Colony stimulating factor 2 (granulocyte-macrophage)	NM_009969
105	Ctsb	Cathepsin B	NM_007798
106	Ctse	Cathepsin E	NM_007799
107	Cyc1	Cytochrome c-1	NM_025567
108	Cyld	Cylindromatosis (turban tumor syndrome)	NM_173369
109	Cyp1a1	Cytochrome P450, family 1, subfamily a, polypeptide 1	NM_009992
110	Cyp1a2	Cytochrome P450, family 1, subfamily a, polypeptide 2	NM_009993
111	Cyp2d22	Cytochrome P450, family 2, subfamily d, polypeptide 22	NM_019823
112	Cyp2e1	Cytochrome P450, family 2, subfamily e, polypeptide 1	NM_021282
113	Cyp3a11	Cytochrome P450, family 3, subfamily a, polypeptide 11	NM_007818
114	Cyp4a14	Cytochrome P450, family 4, subfamily a, polypeptide 14	NM_007822
115	Cyp7a1	Cytochrome P450, family 7, subfamily a, polypeptide 1	NM_007824
116	Cyp7b1	Cytochrome P450, family 7, subfamily b, polypeptide 1	NM_007825
117	Ddit3	DNA-damage inducible transcript 3	NM_007837
118	Defb1	Defensin beta 1	NM_007843
119	Derl1	Der1-like domain family, member 1	NM_024207
120	Dhcr24	24-dehydrocholesterol reductase	NM_053272
121	Dlat	Dihydrolipoamide S-acetyltransferase (E2 component of pyruvate dehydrogenase complex)	NM_145614
122	Dld	Dihydrolipoamide dehydrogenase	NM_007861
123	Dlst	Dihydrolipoamide S-succinyltransferase (E2 component of 2-oxo-glutarate complex)	NM_030225
124	Dnaja1	DnaJ (Hsp40) homolog, subfamily A, member 1	NM_008298
125	Dnaja2	DnaJ (Hsp40) homolog, subfamily A, member 2	NM_019794
126	Dnaja3	DnaJ (Hsp40) homolog, subfamily A, member 3	NM_023646
127	Dnajb1	DnaJ (Hsp40) homolog, subfamily B, member 1	NM_018808
128	Dnajb6	DnaJ (Hsp40) homolog, subfamily B, member 6	NM_011847
129	Dnajc3	DnaJ (Hsp40) homolog, subfamily C, member 3	NM_008929
130	Dnajc5	DnaJ (Hsp40) homolog, subfamily C, member 5	NM_016775
131	Dnajc6	DnaJ (Hsp40) homolog, subfamily C, member 6	NM_198412
132	Dnm1	Dynamin 1	NM_010065
133	Dpysl4	Dihydropyrimidinase-like 4	NM_011993
134	Duox1	Dual oxidase 1	NM_001099297
135	Duox2	Dual oxidase 2	NM_177610
136	Echs1	Enoyl Coenzyme A hydratase, short chain, 1, mitochondrial	NM_053119
137	Edem1	ER degradation enhancer, mannosidase alpha-like 1	NM_138677
138	Edem3	ER degradation enhancer, mannosidase alpha-like 3	NM_001039644
139	Ehhadh	Enoyl-Coenzyme A, hydratase/3-hydroxyacyl Coenzyme A dehydrogenase	NM_023737
140	Eif2ak3	Eukaryotic translation initiation factor 2 alpha kinase 3	NM_010121

141	Eif5b	Eukaryotic translation initiation factor 5B	NM_198303
142	Eno1	Enolase 1, alpha non-neuron	NM_023119
143	Ep300	E1A binding protein p300	NM_177821
144	Ephx1	Epoxide hydrolase 1, microsomal	NM_010145
145	Epx	Eosinophil peroxidase	NM_007946
146	Ercc1	Excision repair cross-complementing rodent repair deficiency, complementation group 1	NM_007948
147	Ercc2	Excision repair cross-complementing rodent repair deficiency, complementation group 2	NM_007949
148	Ercc3	Excision repair cross-complementing rodent repair deficiency, complementation group 3	NM_133658
149	Ercc5	Excision repair cross-complementing rodent repair deficiency, complementation group 5	NM_011729
150	Ercc6	Excision repair cross-complementing rodent repair deficiency, complementation group 6	NM_001081221
151	Ern2	Endoplasmic reticulum (ER) to nucleus signalling 2	NM_012016
152	Ero1l	ERO1-like (S. cerevisiae)	NM_015774
153	Ero1lb	ERO1-like beta (S. cerevisiae)	NM_026184
154	Esd	Esterase D/formylglutathione hydrolase	NM_016903
155	Esr1	Estrogen receptor 1 (alpha)	NM_007956
156	F2	Coagulation factor II	NM_010168
157	Fabp1	Fatty acid binding protein 1, liver	NM_017399
158	Fadd	Fas (TNFRSF6)-associated via death domain	NM_010175
159	Fas	Fas (TNF receptor superfamily member 6)	NM_007987
160	Fasl	Fas ligand (TNF superfamily, member 6)	NM_010177
161	Fasn	Fatty acid synthase	NM_007988
162	Fbxo6	F-box protein 6	NM_015797
163	Fh1	Fumarate hydratase 1	NM_010209
164	Fmo2	Flavin containing monooxygenase 2	NM_018881
165	Fmo3	Flavin containing monooxygenase 3	NM_008030
166	Fmo4	Flavin containing monooxygenase 4	NM_144878
167	Fmo5	Flavin containing monooxygenase 5	NM_010232
168	Foxi1	Forkhead box I1	NM_023907
169	Timm10b	Fractured callus expressed transcript 1	NM_019502
170	Gadd45a	Growth arrest and DNA-damage-inducible 45 alpha	NM_007836
171	Galnt5	UDP-N-acetyl-alpha-D-galactosamine:polypeptide N-acetylgalactosaminyltransferase 5	NM_172855
172	Gcdh	Glutaryl-Coenzyme A dehydrogenase	NM_008097
173	Gpd1	Glycerol-3-phosphate dehydrogenase 1 (soluble)	NM_010271
174	Gpt	Glutamic pyruvic transaminase, soluble	NM_182805
175	Gpx1	Glutathione peroxidase 1	NM_008160
176	Gpx2	Glutathione peroxidase 2	NM_030677
177	Gpx3	Glutathione peroxidase 3	NM_008161
178	Gpx4	Glutathione peroxidase 4	NM_008162
179	Gpx5	Glutathione peroxidase 5	NM_010343
180	Gpx6	Glutathione peroxidase 6	NM_145451
181	Gpx7	Glutathione peroxidase 7	NM_024198
182	Grb2	Growth factor receptor bound protein 2	NM_008163
183	Gsta3	Glutathione S-transferase, alpha 3	NM_010356
184	Gstm4	Glutathione S-transferase, mu 4	NM_026764
185	H2-Eb1	Histocompatibility 2, class II antigen E beta	NM_010382
186	Vimp	Histocompatibility 47	NM_024439

187	Haao	3-hydroxyanthranilate 3,4-dioxygenase	NM_025325
188	Hadha	Hydroxyacyl-Coenzyme A dehydrogenase/3-ketoacyl-Coenzyme A thiolase/enoyl-Coenzyme A hydratase (trifunctional protein), alpha subunit	NM_178878
189	Hadhb	Hydroxyacyl-Coenzyme A dehydrogenase/3-ketoacyl-Coenzyme A thiolase/enoyl-Coenzyme A hydratase (trifunctional protein), beta subunit	NM_145558
190	Herpud1	Homocysteine-inducible, endoplasmic reticulum stress-inducible, ubiquitin-like domain member 1	NM_022331
191	Hpn	Hepsin	NM_008281
192	Hpx	Hemopexin	NM_017371
193	Hrg	Histidine-rich glycoprotein	NM_053176
194	Hsf1	Heat shock factor 1	NM_008296
195	Hsf2	Heat shock factor 2	NM_008297
196	Hsp90aa1	Heat shock protein 90, alpha (cytosolic), class A member 1	NM_010480
197	Hsp90ab1	Heat shock protein 90 alpha (cytosolic), class B member 1	NM_008302
198	Hspa1a	Heat shock protein 1A	NM_010479
199	Hspa1l	Heat shock protein 1-like	NM_013558
200	Hspa2	Heat shock protein 2	NM_008301
201	Hspa4	Heat shock protein 4	NM_008300
202	Hspa5	Heat shock protein 5	NM_022310
203	Hspa8	Heat shock protein 8	NM_031165
204	Hspa9	Heat shock protein 9	NM_010481
205	Hspb1	Heat shock protein 1	NM_013560
206	Hspb2	Heat shock protein 2	NM_024441
207	Hspb6	Heat shock protein, alpha-crystallin-related, B6	NM_001012401
208	Hspb7	Heat shock protein family, member 7 (cardiovascular)	NM_013868
209	Hspb8	Heat shock protein 8	NM_030704
210	Hspbap1	Hspb associated protein 1	NM_175111
211	Hspd1	Heat shock protein 1 (chaperonin)	NM_010477
212	Hspe1	Heat shock protein 1 (chaperonin 10)	NM_008303
213	Hsph1	Heat shock 105kDa/110kDa protein 1	NM_013559
214	Htra2	HtrA serine peptidase 2	NM_019752
215	Htra4	HtrA serine peptidase 4	NM_001081187
216	Icam1	Intercellular adhesion molecule 1	NM_010493
217	Idh1	Isocitrate dehydrogenase 1 (NADP+), soluble	NM_010497
218	Idh2	Isocitrate dehydrogenase 2 (NADP+), mitochondrial	NM_173011
219	Idh3a	Isocitrate dehydrogenase 3 (NAD+) alpha	NM_029573
220	Idh3b	Isocitrate dehydrogenase 3 (NAD+) beta	NM_130884
221	Idh3g	Isocitrate dehydrogenase 3 (NAD+), gamma	NM_008323
222	Ifng	Interferon gamma	NM_008337
223	Il10	Interleukin 10	NM_010548
224	Il13	Interleukin 13	NM_008355
225	Il1a	Interleukin 1 alpha	NM_010554
226	Il1b	Interleukin 1 beta	NM_008361
227	Il2	Interleukin 2	NM_008366
228	Il2ra	Interleukin 2 receptor, alpha chain	NM_008367
229	Il4	Interleukin 4	NM_021283
230	Il5	Interleukin 5	NM_010558
231	Il6	Interleukin 6	NM_031168
232	Inhbe	Inhibin beta E	NM_008382
233	Irf6	Interferon regulatory factor 6	NM_016851

234	Itgax	Integrin alpha X	NM_021334
235	Jag1	Jagged 1	NM_013822
236	Jph3	Junctophilin 3	NM_020605
237	Kcnip1	Kv channel-interacting protein 1	NM_027398
238	Khk	Ketohexokinase	NM_008439
239	Klf1	Kruppel-like factor 1 (erythroid)	NM_010635
240	Lig4	Ligase IV, DNA, ATP-dependent	NM_176953
241	Lmna	Lamin A	NM_019390
242	Lpl	Lipoprotein lipase	NM_008509
243	Lss	Lanosterol synthase	NM_146006
244	Ly6d	Lymphocyte antigen 6 complex, locus D	NM_010742
245	Lyz2	Lysozyme 2	NM_017372
246	Mag	Myelin-associated glycoprotein	NM_010758
247	Manba	Mannosidase, beta A, lysosomal	NM_027288
248	Maoa	Monoamine oxidase A	NM_173740
249	Maob	Monoamine oxidase B	NM_172778
250	Mapk8	Mitogen-activated protein kinase 8	NM_016700
251	Mbtps1	Membrane-bound transcription factor peptidase, site 1	NM_019709
252	Mbtps2	Membrane-bound transcription factor peptidase, site 2	NM_172307
253	Mcl1	Myeloid cell leukemia sequence 1	NM_008562
254	Mdh1	Malate dehydrogenase 1, NAD (soluble)	NM_008618
255	Mdh1b	Malate dehydrogenase 1B, NAD (soluble)	NM_029696
256	Mdh2	Malate dehydrogenase 2, NAD (mitochondrial)	NM_008617
257	Mdm2	Transformed mouse 3T3 cell double minute 2	NM_010786
258	Metap2	Methionine aminopeptidase 2	NM_019648
259	Mgmt	O-6-methylguanine-DNA methyltransferase	NM_008598
260	Mki67	Antigen identified by monoclonal antibody Ki 67	NM_001081117
261	Mlh1	MutL homolog 1 (E. coli)	NM_026810
262	Mlx	MAX-like protein X	NM_011550
263	Mpo	Myeloperoxidase	NM_010824
264	Mrps18b	Mitochondrial ribosomal protein S18B	NM_025878
265	Msh2	MutS homolog 2 (E. coli)	NM_008628
266	Mttp	Microsomal triglyceride transfer protein	NM_008642
267	Nfkb1	Nuclear factor of kappa light polypeptide gene enhancer in B-cells 1, p105	NM_008689
268	Nploc4	Nuclear protein localization 4 homolog (S. cerevisiae)	NM_199469
269	Nqo1	NAD(P)H dehydrogenase, quinone 1	NM_008706
270	Nr0b2	Nuclear receptor subfamily 0, group B, member 2	NM_011850
271	Nr1h4	Nuclear receptor subfamily 1, group H, member 4	NM_009108
272	Nr5a2	Nuclear receptor subfamily 5, group A, member 2	NM_030676
273	Nucb1	Nucleobindin 1	NM_008749
274	Nudt1	Nudix (nucleoside diphosphate linked moiety X)-type motif 1	NM_008637
275	Nudt13	Nudix (nucleoside diphosphate linked moiety X)-type motif 13	NM_026341
276	Nudt15	Nudix (nucleoside diphosphate linked moiety X)-type motif 15	NM_172527
277	Nup210	Nucleoporin 210	NM_018815
278	Ogdhl	Oxoglutarate dehydrogenase-like	NM_001081130
279	Ogg1	8-oxoguanine DNA-glycosylase 1	NM_010957
280	Olfr1404	Olfactory receptor 1404	NM_146881
281	Os9	Amplified in osteosarcoma	NM_177614

282	Slc51a	Organic solute transporter alpha	NM_145932
283	Slc51b	Organic solute transporter beta	NM_178933
284	Parp1	Poly (ADP-ribose) polymerase family, member 1	NM_007415
285	Parp2	Poly (ADP-ribose) polymerase family, member 2	NM_009632
286	Pcca	Propionyl-Coenzyme A carboxylase, alpha polypeptide	NM_144844
287	Pcna	Proliferating cell nuclear antigen	NM_011045
288	Pdyn	Prodynorphin	NM_018863
289	Pfdn5	Prefoldin 5	NM_027044
290	Pnpla3	Patatin-like phospholipase domain containing 3	NM_054088
291	Pon1	Paraoxonase 1	NM_011134
292	Por	P450 (cytochrome) oxidoreductase	NM_008898
293	Pou3f3	POU domain, class 3, transcription factor 3	NM_008900
294	Ppara	Peroxisome proliferator activated receptor alpha	NM_011144
295	Pparg	Peroxisome proliferator activated receptor gamma	NM_011146
296	Ppia	Peptidylprolyl isomerase A	NM_008907
297	Ppp1r15b	Protein phosphatase 1, regulatory (inhibitor) subunit 15b	NM_133819
298	Prdx1	Peroxiredoxin 1	NM_011034
299	Prdx2	Peroxiredoxin 2	NM_011563
300	Prdx6	Peroxiredoxin 6	NM_007453
301	Prkdc	Protein kinase, DNA activated, catalytic polypeptide	NM_011159
302	Ptgs2	Prostaglandin-endoperoxide synthase 2	NM_011198
303	Ptprc	Protein tyrosine phosphatase, receptor type, C	NM_011210
304	Pvr	Poliovirus receptor	NM_027514
305	Rab25	RAB25, member RAS oncogene family	NM_016899
306	Rad51	RAD51 homolog (S. cerevisiae)	NM_011234
307	Rdx	Radixin	NM_009041
308	Retn	Resistin	NM_022984
309	S100a7a	S100 calcium binding protein A7A	NM_199422
310	S100a8	S100 calcium binding protein A8 (calgranulin A)	NM_013650
311	Sc4mol	Sterol-C4-methyl oxidase-like	NM_025436
312	Scd1	Stearoyl-Coenzyme A desaturase 1	NM_009127
313	Sdha	Succinate dehydrogenase complex, subunit A, flavoprotein (Fp)	NM_023281
314	Sdhb	Succinate dehydrogenase complex, subunit B, iron sulfur (Ip)	NM_023374
315	Sdhc	Succinate dehydrogenase complex, subunit C, integral membrane protein	NM_025321
316	Sdhd	Succinate dehydrogenase complex, subunit D, integral membrane protein	NM_025848
317	Sec62	SEC62 homolog (S. cerevisiae)	NM_027016
318	Sel1l	Sel-1 suppressor of lin-12-like (C. elegans)	NM_001039089
319	Serp1	Stress-associated endoplasmic reticulum protein 1	NM_030685
320	Serpina3k	Serine (or cysteine) peptidase inhibitor, clade A, member 3K	NM_011458
321	Serpina3n	Serine (or cysteine) peptidase inhibitor, clade A, member 3N	NM_009252
322	Slc10a1	Solute carrier family 10 (sodium/bile acid cotransporter family), member 1	NM_011387
323	Slc2a3	Solute carrier family 2 (facilitated glucose transporter), member 3	NM_011401
324	Slco1a4	Solute carrier organic anion transporter family, member 1a4	NM_030687
325	Smpd1	Sphingomyelin phosphodiesterase 1, acid lysosomal	NM_011421
326	Sod1	Superoxide dismutase 1, soluble	NM_011434
327	Spata2	Spermatogenesis associated 2	NM_170756
328	Srebf1	Sterol regulatory element binding transcription factor 1	NM_011480
329	Stbd1	Starch binding domain 1	NM_175096

330	Sucla2	Succinate-Coenzyme A ligase, ADP-forming, beta subunit	NM_011506
331	Suclg1	Succinate-CoA ligase, GDP-forming, alpha subunit	NM_019879
332	Suclg2	Succinate-Coenzyme A ligase, GDP-forming, beta subunit	NM_011507
333	Sycp2	Synaptonemal complex protein 2	NM_177191
334	Syt1	Synaptotagmin I	NM_009306
335	Syvn1	Synovial apoptosis inhibitor 1, synoviolin	NM_028769
336	Tagln	Transgelin	NM_011526
337	Tcp1	T-complex protein 1	NM_013686
338	Tff3	Trefoil factor 3, intestinal	NM_011575
339	Tgfb1	Transforming growth factor, beta 1	NM_011577
340	Tmem57	Transmembrane protein 57	NM_025382
341	Tnf	Tumor necrosis factor	NM_013693
342	Tnfaip8l1	Tumor necrosis factor, alpha-induced protein 8-like 1	NM_025566
343	Tnfrsf10b	Tumor necrosis factor receptor superfamily, member 10b	NM_020275
344	Tnfrsf1a	Tumor necrosis factor receptor superfamily, member 1a	NM_011609
345	Tnfsf10	Tumor necrosis factor (ligand) superfamily, member 10	NM_009425
346	Tpo	Thyroid peroxidase	NM_009417
347	Trim10	Tripartite motif-containing 10	NM_011280
348	Trp53	Transformation related protein 53	NM_011640
349	Txnip	Thioredoxin interacting protein	NM_023719
350	Txnl4b	Thioredoxin-like 4B	NM_175646
351	Txnrd2	Thioredoxin reductase 2	NM_013711
352	Ube2g2	Ubiquitin-conjugating enzyme E2G 2	NM_019803
353	Ube2j2	Ubiquitin-conjugating enzyme E2, J2 homolog (yeast)	NM_001039157
354	Ubqln2	Ubiquilin 2	NM_018798
355	Ubxn4	UBX domain protein 4	NM_026390
356	Ucp1	Uncoupling protein 1 (mitochondrial, proton carrier)	NM_009463
357	Ucp2	Uncoupling protein 2 (mitochondrial, proton carrier)	NM_011671
358	Ucp3	Uncoupling protein 3 (mitochondrial, proton carrier)	NM_009464
359	Ugt1a1	UDP glucuronosyltransferase 1 family, polypeptide A1	NM_201645
360	Ugt2a1	UDP glucuronosyltransferase 2 family, polypeptide A1	NM_053184
361	Ugt2b1	UDP glucuronosyltransferase 2 family, polypeptide B1	NM_152811
362	Uox	Urate oxidase	NM_009474
363	Vcp	Valosin containing protein	NM_009503
364	Wipi1	WD repeat domain, phosphoinositide interacting 1	NM_145940
365	Xbp1	X-box binding protein 1	NM_013842
366	Xiap	X-linked inhibitor of apoptosis	NM_009688
367	Xpa	Xeroderma pigmentosum, complementation group A	NM_011728
368	Xpc	Xeroderma pigmentosum, complementation group C	NM_009531
369	Xrcc1	X-ray repair complementing defective repair in Chinese hamster cells 1	NM_009532
370	Xrcc5	X-ray repair complementing defective repair in Chinese hamster cells 5	NM_009533
